# A U-Net Based Approach for Automating Tribological Experiments

**DOI:** 10.3390/s20226703

**Published:** 2020-11-23

**Authors:** Benjamin Staar, Suleyman Bayrak, Dominik Paulkowski, Michael Freitag

**Affiliations:** 1 BIBA—Bremer Institut für Produktion und Logistik GmbH, University of Bremen, Hochschulring 20, 28359 Bremen, Germany; fre@biba.uni-bremen.de; 2 University of Bremen, Faculty of Production Engineering, Badgasteiner Straße 1, 28359 Bremen, Germany; 3 Fraunhofer Institute for Manufacturing Technology and Advanced Materials IFAM, Wiener Strasse 12, 28359 Bremen, Germany; suleyman.bayrak@ifam.fraunhofer.de (S.B.); dominik.paulkowski@ifam.fraunhofer.de (D.P.)

**Keywords:** convolutional neural network, tribology, semantic segmentation

## Abstract

Tribological experiments (i.e., characterizing the friction and wear behavior of materials) are crucial for determining their potential areas of application. Automating such tests could hence help speed up the development of novel materials and coatings. Here, we utilize convolutional neural networks (CNNs) to automate a common experimental setup whereby an endoscopic camera was used to measure the contact area between a rubber sample and a spherical counterpart. Instead of manually determining the contact area, our approach utilizes a U-Net-like CNN architecture to automate this task, creating a much more efficient and versatile experimental setup. Using a 5× random permutation cross validation as well as additional sanity checks, we show that we approached human-level performance. To ensure a flexible and mobile setup, we implemented the method on an NVIDIA Jetson AGX Xavier development kit where we achieved ~18 frames per second by employing mixed-precision training.

## 1. Introduction

**Figure 1:**
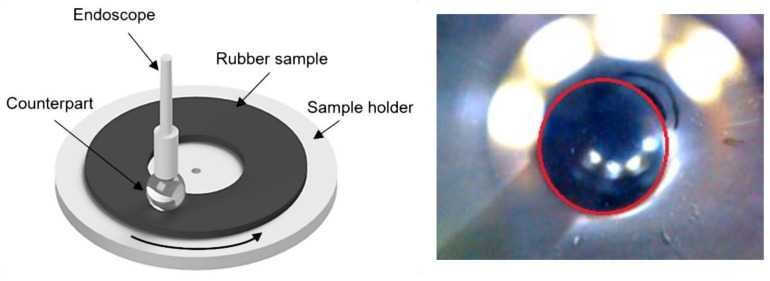
(**Left**) Experimental setup for measuring wear behavior of rubber samples. An endoscopic camera was used to detect changes in the contact area due to material wear. (**Right**) Sample image acquired with the setup. The dark sphere in the center is the contact area (marked with a red circle). It can be seen that multiple reflections are present, increasing the difficulty for contact area segmentation.

Tribology studies the interaction of surfaces in relative motion (i.e., friction and wear behavior of different parts in a mechanical system). For example, the performance of bearings or sealings are mainly controlled by the tribology. Tribological tests hence provide crucial insights into the friction and wear behavior of different materials under laboratory conditions. There exists a multitude of different test setups to investigate tribological behavior. Here, we focused on one common setup whereby an endoscopic camera is used to investigate the contact area between a rubber sample and a spherical counterpart (see Figure 1). The contact area is highly dependent on the viscoelastic properties of the material [1]. Surface properties like adhesion are hence directly related to the contact area [2]. Currently, the evaluation is done manually (i.e., the contact area is marked by hand) in selected frames of the resulting video. This is a tedious process that slows down und hence limits the experiments.

Here, we automated this process using recent advances in semantic segmentation based on deep neural networks. For evaluation, we used two commonly used metrics for semantic segmentation (i.e., the mean intersection over union (mIoU) and the Sørensen–Dice coefficient (SDC)). Utilizing a U-Net [3] based network architecture with a pre-trained residual network [4], we consistently achieved mIoU > 0.92 and SDC > 0.96 on a manually labelled dataset, approaching human level performance. Furthermore, we confirmed the applicability of our approach in several sanity checks. To ensure a flexible and mobile setup, we implemented the method on an NVIDIA Jetson AGX Xavier (NVIDIA, Santa Clara, CA, USA) development kit. By utilizing mixed-precision training [5], we achieved close to real time evaluation with a frame rate of ~18 frames per second. Our method allows for a much more fine-grained temporal sampling of the changes in contact area size while freeing up valuable human resources. This not only allows for a more detailed analysis of wear behavior over time, but opens up the possibility of more elaborate experiments involving feedback loops, like changing the rotation speed or temperature depending on abrasion, possibly counteracting wear, and prolonging components lifetimes. The methods we applied here draw from recent developments in competitive data science settings and are likely to also benefit other new areas of applications that are still without existing benchmark datasets.

### State of the Art

Convolutional neural networks (CNN) are the backbone for many improvements in image processing over the recent decade and are hence the key component in state-of-the-art solutions for popular benchmark datasets like ImageNet [6,7,8] or MS COCO [9,10,11]. This holds true for semantic segmentation (i.e., the pixel-wise labeling of input images). CNN-based algorithms are the top performing solutions for the PASCAL VOC 2012 [12,13,14] dataset, cityscapes [15,16,17], and ADE20K [10,18,19]. There have also been multiple proposals for using CNNs to analyze endoscopic camera images, predominantly in the medical field [20,21,22].

Currently, the application of CNNs in tribological research is mostly focused on defect (i.e., wear detection). For example, Wen et al. [23] used CNNs to implement visual surface inspection of bearing rollers. Pen et al. [24] classified wear particles using a combination of CNNs and support vector machines (SVM). Chang et al. [25] successfully applied CNNs to the automatic detection and severity assessment of gear wear.

However, there have also been approaches using computer vision to aid quantitative analysis of wear behavior. For example, Yu et al. [26] used background subtraction methods to segment wear particles, allowing them to quantify the wear of pivot bearings. Liu et al. [27] used the JPEG segmentation algorithm (JSEG) [28] to segment wear particles in ferrographic images. More recently, Wang et al. [29] used CNNs to classify, quantify, and register wear debris. To the best of our knowledge, using CNN-based image segmentation techniques to quantify changes in contact area in tribological experiments is a novel approach that has not been presented before.

## 2. Materials and Methods

### 2.1. Setup

Figure 1 shows the setup that was automated in this work. Using an endoscopic camera, the contact area between a rubber sample and a spherical counterpart with a diameter of 10 mm^2^ was examined. Each experiment consisted of three stages. In stage 1, the spherical counterpart is slowly lowered into the rubber sample. In stage 2, the rotary table is spun for a predetermined amount of time to create friction. In stage 3, the spherical counterpart is removed from the rubber sample. The aim of the experiment was to document material wear during stage 2. This is accomplished by evaluating changes in the contact area. At the beginning, the contact area measured approximately 6.7 mm^2^. This contact area segmentation is currently done manually, which only allows for sparse temporal sampling. By automating contact area segmentation, it becomes possible to obtain fine-grained (i.e., frame-wise information about changes in contact area) sampling in a shorter amount of time while freeing up human resources.

### 2.2. Dataset

Our dataset consisted of 802 RGB images with 640 × 480 resolution extracted from 40 videos recorded with different materials at different temperatures. However, in this work, we downsampled the resolution to 320 × 240 as processing larger resolution images greatly increases the training and inference times with little to no expected benefit; due to motion blur, reflections, and noise, manual labels are subject to variance (more details in Section 2.7). The performance gain of using the full resolution images was expected to be negligible.

Contact area masks were created by two different annotators. One annotator labeled the data connecting a series of lines (i.e., the “Lasso”-tool), while one annotator drew ellipses that fit the estimated contact area as close as possible. Sample images with corresponding segmentation masks for the contact area are shown in Figure 1. Since the labels were rather coarse, we also used three videos to execute sanity checks (i.e., test if the results matched our expectations/observations; a detailed description is provided in Section 2.7).

### 2.3. Pre-Processing and Data Augmentation

**Figure 2:**
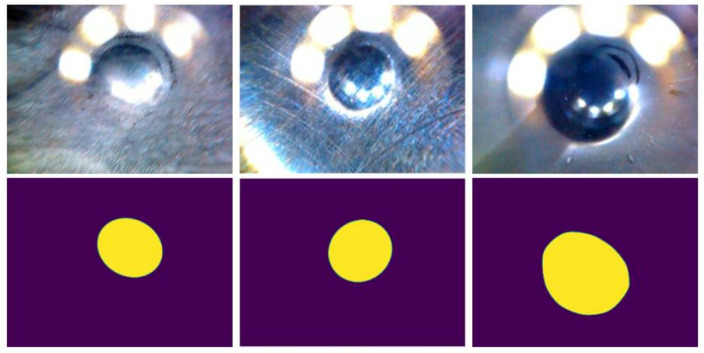
(**Top**) Sample images with different surface coatings and bearing area sizes. (**Bottom**) Corresponding bearing area masks.

In order to possibly improve detection, we also created a pre-processed dataset where we added temporal information by replacing the red color channel of each image with the grayscaled first frame of the corresponding video (see Figure 2). Thus, the difference between the first frame and the respective image is highlighted, often corresponding to the contact area.

**Figure 3:**
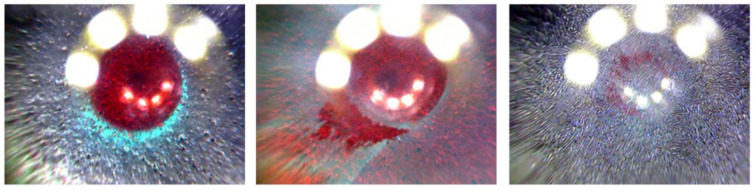
Sample images of pre-processed dataset, where the red color channel is replaced with the first frame of the corresponding video. (**Left**) Ideal case where only the contact area is highlighted. (**Middle**) Wear particles are also marked. (**Right**) Certain coatings diminish the effects of pre-processing.

Ideally, taking the difference between the current and the first frame would be sufficient to automatically segment the bearing area, however, as can be seen in Figure 3, multiple fringe cases, like the occurrence of wear particles or the effect of different coatings, arise during application, which would have to be solved by human intervention.

**Figure 4:**
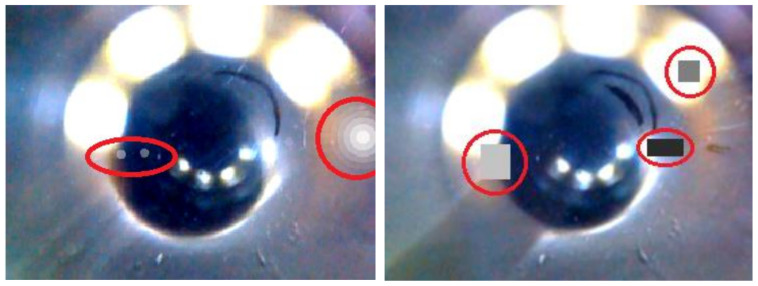
Examples for the data augmentation techniques used in this work (highlighted by red circles). (**Left**) RandomSunFlare as provided by the albumentations library with settings adjusted to fit our needs. (**Right**) Modified version of cutout data augmentation technique to coarsely simulate additional lights and debris.

For data augmentation (i.e., to artificially increase the amount of training data) we used image rotations within a range of ±10° as well as brightness and contrast variations as implemented in the fast.ai library [30]. Additionally, we added several methods from the albumentations library [31], namely contrast limited adaptive histogram equalization (CLAHE), decreasing JPEG compression (ImageCompression), camera sensor noise (ISONoise) as well as simulating sun flares (RandomSunFlare) (see Figure 4, left). For all but the latter method, we used the default settings. For random sun flares, we decreased the source radius to one hundred to better emulate the lighting effects present in the dataset. One of the main problems of the dataset were reflections, debris, and lighting. In order to increase the network’s robustness against these issues, we added a modified version of cutout [32], whereby random rectangular parts of the image are “cut-out” (i.e., replaced by a given fill value). In our modified version, we randomly switched between fill values between the lower and the upper limit of the image pixel value range, coarsely simulating additional lights and debris (Figure 4, right).

### 2.4. U-Net Architecture with Pre-Trained Head

**Figure 5:**
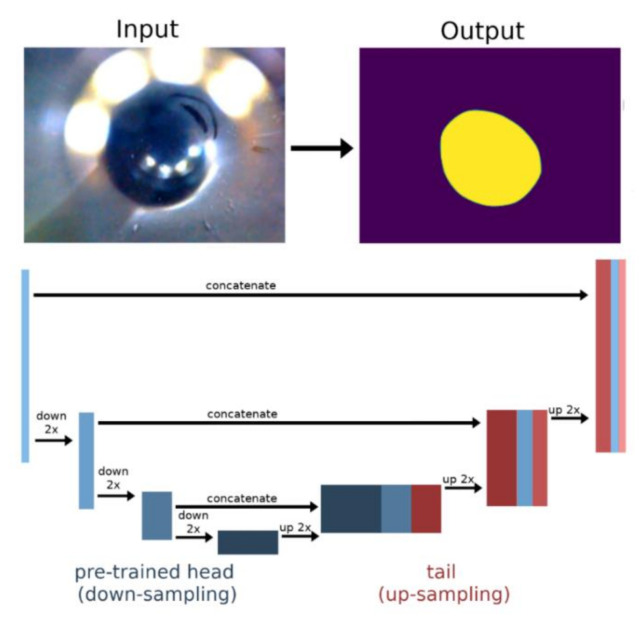
Schematic of the U-Net architecture. Bars indicates blocks of layers with the same spatial resolution: bar height illustrates spatial resolution, bar width the number of features. The input image was fed into the pre-trained network (left), whereby the spatial resolution was reduced while the number of features was increased. Spatial resolution was then restored by up-sampling low-spatial-resolution features, concatenating them with the corresponding higher-resolution features and integrating the results with new additional layers (tail). The number of down- and up-sampling steps depends on the architecture of the pre-trained head.

For our experiments, we used the *unet_learner* function as provided by the fast.ai library with the standard implementation for a U-Net [3] with a pre-trained head. As additional tweaks, we changed the activation function and added a post-processing filter after the output (see Section 2.5 for details). The network architecture is illustrated in Figure 5. The incoming image is first processed by the network’s head, which in our case consisted of a pre-trained CNN. Thereby, the spatial resolution is decreased while the number of features is increased, a standard procedure adopted in almost all modern convolutional neural networks. Since we aimed for a pixel-wise classification, the spatial resolution needed to be restored. This was achieved by augmenting the network with an up-sampling part. A key feature of the U-Net architecture is the use of skip connections that combine high-level, low-resolution features with lower-level, higher-resolution features. Thereby, the output of a deeper (i.e., high-level) layer is up-sampled to match the spatial resolution of its preceding layer. The features are then concatenated and combined using additional convolutional layers.

The fast.ai implementation, aside from simplifying the use of pre-trained networks, increases the efficiency of the architecture by replacing the default up-sampling operation with pixel shuffling [33].

In our work, we tested two different pre-trained residual networks, ResNet18 and ResNet34 [4], since they provide a good trade-off between discriminative power and computation speed. Using models trained on one dataset to train a model for a novel dataset is called transfer learning [34] and has been shown to benefit a variety of image-processing tasks. Usually, pre-training is conducted on the ImageNet dataset [6], which is also the case in our experiments. As above-mentioned, we compared ResNet18 and ResNet34 whereby the respective number corresponded to the network’s depth and hence complexity.

### 2.5. Network Tweaks

The pre-trained models utilized in this work used the rectified linear unit (ReLU) as activation function [35], as in most fundamental research on CNNs. In academic research, this ensures comparability with previous work (if the focus of a paper is not introducing a novel activation function). However, multiple alternatives have been proposed that alleviate problems associated with ReLU (i.e., the dying ReLU-problem) and generally improve performance. One of these alternatives is the parametric ReLU (PReLU) activation function [36], which generalizes (i.e., includes) the ReLU function while keeping the benefit of a low computational cost:

$$\mathrm{PReLU}\left(x\right)=\;\left\{\begin{array}{c} x,\;\mathrm{if}\;x\geq 0\\ \alpha x,\;\mathrm{otherwise} \end{array}\right.$$

The learnable parameter *α* of the PReLU function is thereby flexible (i.e., it can be a single value that is shared by all channels or a vector with separate values for each channel). Here, we opted for the latter (i.e., a trainable value α for each channel). We replaced each ReLU activation function in the pre-trained head of the model with the PReLU activation function with initial values of *α* set to zero, effectively emulating the ReLU activation function at the start of training. In the additional layers (i.e., the networks tail), we initialized *α* with 0.25.

Another tweak to our network was the addition of post-processing in the form of applying a 7 × 7 median filter to the output. This way, the output image is smoothed and outliers (i.e., small numbers of incorrectly labelled pixels) are removed. The median filter was added directly as the last layer of the networks using the Kornia library [37].

### 2.6. Optimization

**Figure 6:**
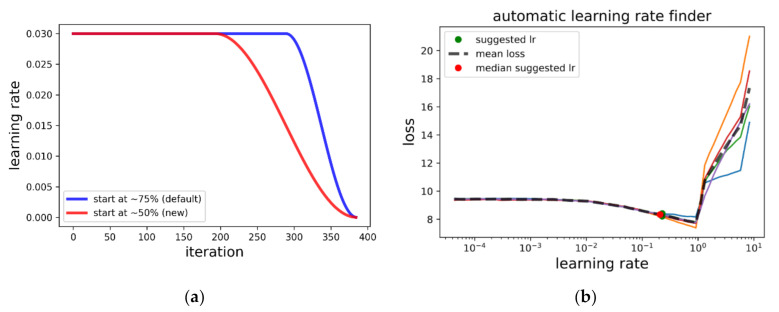
(**a**) Flat + cosine annealing (fca) learning rate schedule. The learning rate is kept steady for a certain percentage of the iterations and decreased afterwards. (**b**) Finding the optimal learning rate: the learning rate is increased at every iteration (batch) while the loss is monitored. The optimal learning rate range shows a steep decrease in loss, followed by a sudden increase.

We used the Ranger optimizer [38] (i.e., a combination of Rectified Adam [39], LookAhead [40] and Gradient Centralization) [41]. According to the recommendations for using Ranger, we used a flat + cosine annealing (fca) learning rate schedule, whereby the learning rate was kept constant for 75% of the training epochs and then decreased via cosine annealing (see Figure 6a for an example). However, in our case, we found that this training policy regularly led to unstable training (i.e., sudden and strong divergence in loss), resulting in very poor models. In order to alleviate this problem, we started the annealing part earlier (i.e., before the epoch where we usually started observing the unstable behavior). We thus started annealing at 50% of the training epochs, resulting in much more stable training behavior.

In order to increase inference speed, we employed mixed-precision training [5] as implemented in the fast.ai library. The resulting models therefore used half- instead of full-precision computations, allowing for significant speedup when using suitable hardware such as the NVIDIA Jetson AGX Xavier used in this work.

As loss function, we combined the Sørensen–Dice coefficient (SDC) [42,43] and the binary cross entropy (BCE):

(1) $$\mathrm{SDC}=\frac{2\mathrm{TP}}{2\mathrm{TP}+\mathrm{FP}+\mathrm{FN}}$$

(2) BCE=−(ylog(p)+(1−y)log(1−p))

(3) Loss=10(1−SDC)+BCE

where TP is the number of true positives; FP is the number of false positives; FN is the number of false negatives; *y* is the target; and *p* is the predicted value. TP, FP, and FN are all calculated with respect to pixel values. By adding BCE to SDC, we removed the occasional convergence issues we encountered by training with pure SDC loss. Since SDC is bounded between zero and one, the training tends to be dominated by the BCE term, especially at the beginning of training. To alleviate this problem, we increased the SDC term by one order of magnitude. Using weighted combinations of SDC and BCE is a commonly used tweak in competitive data science settings (e.g., on www.kaggle.com). It can be viewed as a less sophisticated version of Combo Loss [44] or exponential logarithmic loss [45], which also combines SDC and BCE.

Initially, each model was trained for 10 epochs with fixed pre-trained weights (head). This was followed by an additional 20 epochs of finetuning with a trainable head, whereby the initial learning rate was decreased by two orders of magnitude. We found that training for more epochs only increased the risk for unstable training (i.e., some folds diverging to zero SDC and mIoU) without improvements in the results. In summary, we used the following training schedule for each model:

- 20 epochs: only tail weights trainable, fca schedule, maximum learning rate = $l_{max}$
- 20 epochs: all weights trainable, fca schedule, maximum learning rate = $l_{max}\times \;\mathrm{0.01}$

The learning rates were determined using the learning rate finder proposed by Leslie N. Smith [46] as implemented in the fast.ai library v1. An illustration is provided in Figure 6b: before training, a test is run whereby the learning rate is increased for each batch starting at a very low value of 1 × 10^−7^. The resulting loss vs. learning rate curve is then plotted. The curve usually shows a steady loss for low learning rates, a decrease for the optimal learning rate range, and a steep increase for learning rates that are too high. Through empirical tests, we found that learning rates in the mid-range of the decreasing part yielded the best results for the combination of the U-Net and Ranger optimizer. To obtain robust estimates, we repeated the learning finder five times and took the median suggested learning rate. Using this procedure, we chose an optimal learning rate for each model–parameter combination. The batch size was set to eight for all experiments.

### 2.7. Evaluation

We used the Sørensen–Dice coefficient (SDC) as well as the mean intersection over union (mIoU) as scalar metrics to rate the segmentation performance.

(4) $$\mathrm{mIoU}=\;\frac{\mathrm{TP}}{\mathrm{TP}+\mathrm{FP}+\mathrm{FN}}$$

Again, TP, FP, and FN were all calculated with respect to pixel values. In order to obtain robust estimates of model performance, we used a 5-fold random permutation cross validation for each scenario/parameter setting. Thereby, the data were randomly split five times, with 95% training and 5% validation data and a model was trained and evaluated on each split. The same splits were used for all models to increase comparability. The resulting statistics (i.e., mean and standard deviation) were used for comparison.

One issue when evaluating the model on human-labeled data is that the labels are not perfect (i.e., there is some variance in the labels). In order to get an optimistic estimate on this human-induced label-noise, we had three different people label the same ten images three times. By choosing a small number of images, we tried to exclude the influence of fatigue. All labelers were told to work with great care and that the results would be used to train our model. We then calculated the mean SDC and mIoU between the labels created by different people at different times. We thus calculated a mean SDC of 0.97 ± 0.04 as well as a mean mIoU of 0.94 ± 0.7.

While scalar metrics play an important role in model evaluation, assessing the performance with a single value can hide important aspects that hinder the model’s application. Furthermore, the target segmentation masks used here for training and evaluation were rather coarse, limiting the expressiveness of SDC and mIoU metrics. As an additional sanity check, we hence applied each model to three different videos of experiments with the same material. We then tested if the models correctly predicted similar contact area curves across all three videos. We also tested the repeatability of our approach by evaluating the similarity of the predicted curves across the 5-fold random permutation cross validation. The contact area was thereby calculated by fitting the largest contour in the segmentation mask and calculating the corresponding area. Contour detection was accomplished using the respective function of the OpenCV library [47] for Python (i.e., via the algorithm proposed by Suzuki and Be) [48]. The contour area was calculated by counting the number of pixels inside the largest contour.

### 2.8. Software and Hardware

All experiments were executed using the Python programming language [49]. To implement and train neural networks we used Pytorch [50] and the fast.ai library v1 [30]. All experiments were conducted on a Desktop PC using an NVIDIA Geforce 1080 Ti GPU (NVIDIA, Santa Clara, CA, USA). The algorithm was deployed on an NVIDIA Jetson AGX Xavier development kit.

## 3. Results

### 3.1. Performance

**Figure 7:**
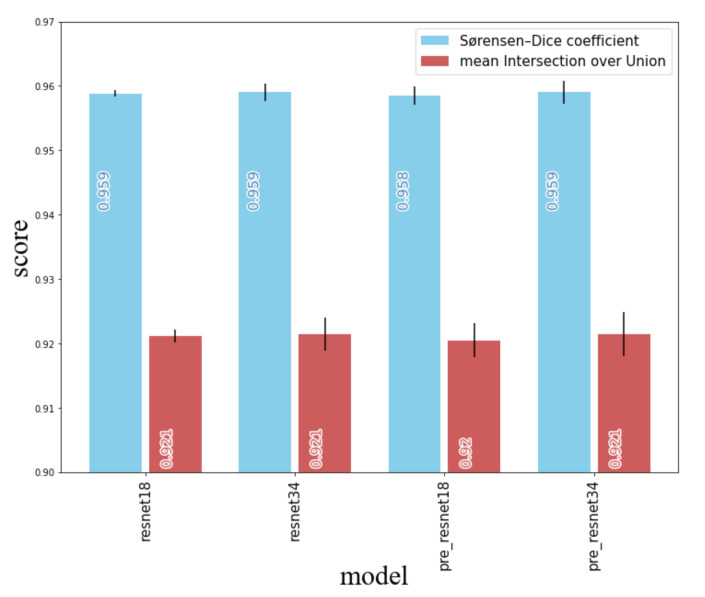
Sørensen–Dice (blue) coefficient as well as mean intersection over union (red) for different pre-trained heads with and without data pre-processing (prefix “pre_”). Scores (rounded to third decimal point) are written into bars.

Figure 7 shows the resulting scores for ResNet18 and ResNet34 with and without pre-processing. It can be seen that increasing the depth of the pre-trained head did not yield any performance gains (i.e., the same results were achieved with both ResNet18 and ResNet34). Pre-processing also did not influence performance.

**Figure 8:**
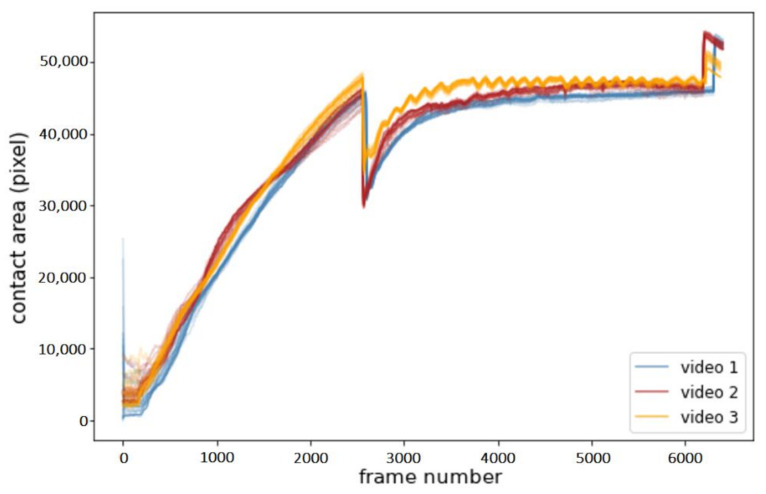
Contact area change over frames for all models trained in this work, color coded by video. Frames 0 to ~2400 show stage 1 (i.e., the spherical counterpart being slowly lowered into the rubber sample). Frame~2400 to ~6000 show stage 2 (i.e., the rotary table being spun to create friction). This is followed by stage 3 (i.e., the spherical counterpart being removed from the sample). The curves for each video were strongly aligned (i.e., model choice has little impact on the resulting contact area curves). Visible differences only occurred at the very beginning as well as at the transition between stages 1 and 2.

Figure 8 shows the example results of the sanity checks (i.e., the predicted contact area for each frame of three test videos). All models produced very similar curves, mirroring the observed similarity in SDC and mIoU. The variance between experiments was thus much larger than the variance introduced by different model and data pre-processing choices.

### 3.2. Failure Cases

**Figure 9:**
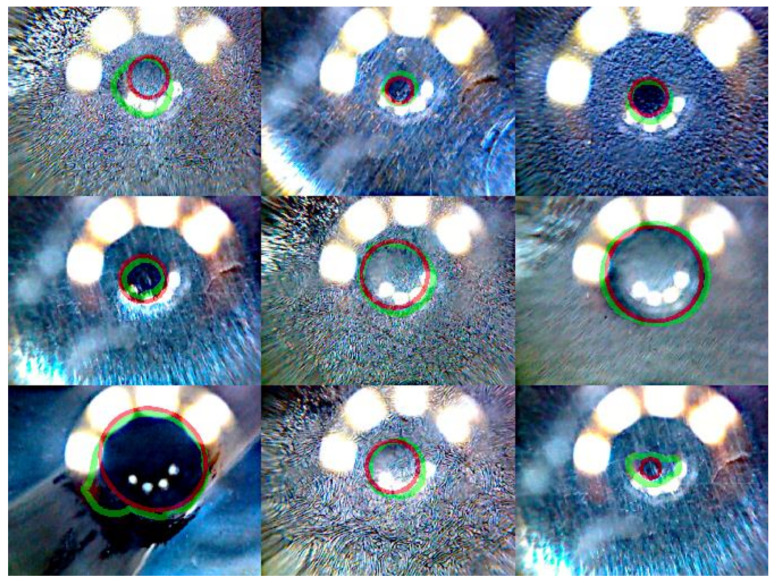
Nine validation samples with the highest loss, when using pre-trained ResNet without pre-processing. Target area is outline in red, predicted area in green.

In order to gain further insight into network performance, we visually examined the validation samples with the highest loss. Figure 9 shows the nine validation samples with the highest loss over all folds when using a pre-trained ResNet18 as the U-Net head without pre-processing.

**Figure 10:**
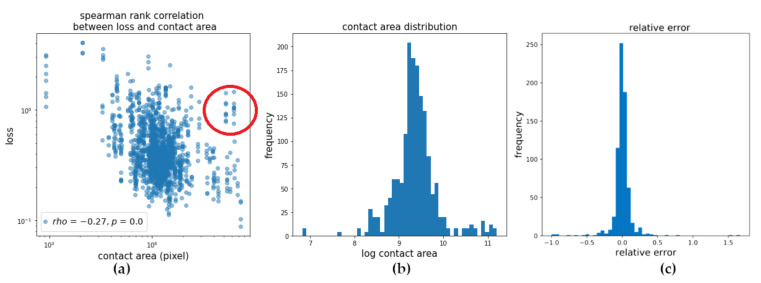
(**a**) Dependency between validation loss and contact area. We found weak, but significant (*p* < 1 × 10^−27^) negative Spearman rank correlation as well as a cluster of high-contact-area samples with comparably high losses (marked by red circle). (**b**) Contact area distribution in the training data. (**c**) Distribution of the relative error between target area and predicted area.

The target area is outlined in red, the predicted area in green. It can be seen that most of the top failure cases came from the initial stage of the experiment (i.e., lowering the spherical counterpart into the rubber sample). This stage starts with a small contact area that increases as the counterpart is lowered. We thus investigated the dependency between contact area and loss. While there was considerable variance, we found significant negative Spearman rank correlation between loss and target area as well as a cluster of high-contact-area samples with comparably high losses (Figure 10a). We also examined if there was a systematic discrepancy between predictions and target values (i.e., if there is a tendency toward under- or over-estimating the contact area with respect to the target values provided by the dataset). We thus calculated the relative error (re) between the predicted and target area:

(5) $$re=\;\frac{A_{p}-A_{t}}{A_{t}}$$

where $A_{p}$ is the predicted area and $A_{t}$ is the target area, both calculated in terms of pixels. The results did not indicate any tendency toward under- or over-estimating the target area.

## 4. Discussion

All models tested in this work reached similar performances of 0.96 SDC and 0.92 mIoU, mirrored by very similar contact area curves when applied to three example videos. Since these were not the best possible values, it seems as if there is some type of performance bottleneck that could not be overcome by using pre-trained residual networks with more layers or applying the aforementioned pre-processing scheme. However, we found that, when applied to various videos (not just the ones used for aforementioned sanity checks), the method produced satisfactory results throughout (i.e., the predicted contact areas closely matched the visually observed contact areas). The likely cause for this observation was imperfect labeling of the training data. Different parts of the data were labeled by different annotators. One annotator labeled the data connecting a series of lines (i.e., the “Lasso”-tool), while one annotator simply drew ellipses that fit the estimated contact area as close as possible. Neither of the techniques are completely accurate. Judging by visual examination when applied to multiple videos, we found that the networks were able to extract the correct contact areas despite this imperfect labeling.

Nevertheless, a few actual failure cases remained and further diminished the results. Looking at the samples with the largest validation loss for all models, we found a prevalence of samples with very low and very high contact areas. Figure 10a shows the dependency between the target contact area and validation loss. There was a significant (*p* < 1 × 10^−27^) negative Spearman rank correlation between the contact area and loss. However, the high variance suggests that there are other important factors at play. Furthermore, there was a cluster of high contact area samples with comparably high loss. Examining the contact area distribution in the dataset, we could identify a likely reason for this, as these cases were less well represented in the data. Adding such samples could hence further improve the quality of the results.

It should also be noted that contact area segmentation, while an important step, is not sufficient to correctly calculate the contact area in terms of mm^2^. In order to fully utilize the setup, we also had to account for image distortions due to the spherical counterpart. This will hence be one of the main areas for future improvements.

Finally, we would like to note, that while there were various fringe cases, contact area segmentation as discussed in this work could likely also be solved by the clever combination of methods from “classical” computer vision. The main advantage of using CNNs is that excellent results can be achieved while the implementation effort shifts from algorithm development toward dataset creation, a task that can be solved by a much broader part of the population. Although we encountered difficulties regarding training stability, we think that as these methods are increasingly applied to industrial problems, more stable default approaches will emerge. Solving computer vision problems would thus become a lot more accessible, without the need for hiring specialists. Therefore, the development of such methods is an important part of our future research.
